# Left-sided Neck Swelling: An Unusual Presentation of a Rare Disease

**DOI:** 10.7759/cureus.4714

**Published:** 2019-05-22

**Authors:** Andrew Charlton

**Affiliations:** 1 Accident and Emergency Department, Bradford Royal Infirmary, Bradford, GBR

**Keywords:** pneumomediastinum, spontaneous pneumomediastinum, chest x-ray (cxr), computed tomography, odynophagia, neck swelling, macklin effect, hamman's sign, subcutaneous emphysema, hamman's syndrome

## Abstract

Pneumomediastinum describes air infiltrating into the soft tissues of the mediastinum. It may be classified as spontaneous or secondary. Spontaneous pneumomediastinum is a rare disease, which usually resolves without intervention. The acute onset of chest pain is the most common presenting complaint.

This article presents a case of spontaneous pneumomediastinum in a 19-year-old man with a short history of rapidly enlarging, painless swelling to the left side of his neck, followed by a discussion of the literature. The diagnosis was made following chest x-ray (CXR) and computed tomography (CT). There was no history of any precipitating event or any risk factors. He was managed conservatively and discharged with outpatient follow-up.

Management of spontaneous pneumomediastinum continues to vary and may benefit from the development of guidelines to standardise management in the future.

## Introduction

Pneumomediastinum refers to the infiltration of air into the mediastinal structures. It is a rare disease with spontaneous mediastinum occurring in approximately one in 30,000 emergency department attendances [1]. It appears to be most common in young men [2].

The diagnosis of pneumomediastinum can be further categorised as spontaneous or secondary according to the cause. Pneumomediastinum is considered secondary when an underlying cause is identified, such as oesophageal perforation, trauma, rupture of a laryngocele, or mediastinitis with gas-forming organisms [3].

Although not completely understood, the mechanism of injury in spontaneous pneumomediastinum is probably by transient elevation of intra-alveolar pressures causing a rupture of the alveoli. Air dissects along the perivascular space in the pulmonary interstitium and into the mediastinum [4]. This is known as the Macklin effect [5]. Spontaneous pneumomediastinum is most commonly associated with a precipitating event, the most common of which are illegal drug consumption, bronchospasm, exercise, and coughing [6].

Spontaneous pneumomediastinum is usually considered a benign disease, although significant complications, such as secondary pneumothorax, extensive subcutaneous emphysema, and compression of major vessels and trachea, have been reported. Investigation and management aim to exclude causes of secondary pneumomediastinum and monitor for complications. Typically, patients recover without requiring intervention and without recurrence [7].

## Case presentation

A 19-year-old man presented to the Emergency Department with a one-day history of rapidly enlarging, painless swelling to the left side of his neck. On presentation, this was his only symptom; however, he subsequently developed odynophagia during his hospital admission. He had no history of trauma, respiratory tract, or gastrointestinal disease. Inspection revealed a swelling of the left side of the neck extending onto the left chest wall. On palpation, this swelling was consistent with subcutaneous emphysema. Observations were all within normal parameters and the patient did not exhibit any signs of respiratory distress.

There was no history of coughing fits, exercise, or other unusual activity. He had no other medical problems. He was not a smoker and did not use recreational drugs. He did not have any other medical problems and there was no history of trauma. There was no family history of inherited disorders nor were there signs suggestive of respiratory disease or connective tissue disorder.

**Figure 1:**
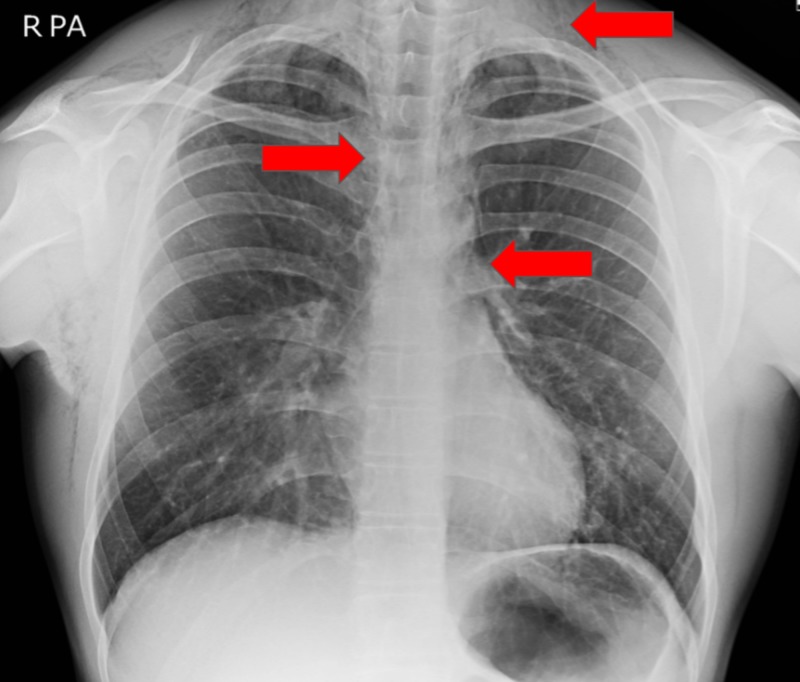
Plain film chest x-ray Pneumomediastinum with widespread air in the mediastinum and extending into the soft tissues of the thorax and neck was noted (red arrows).

**Figure 2:**
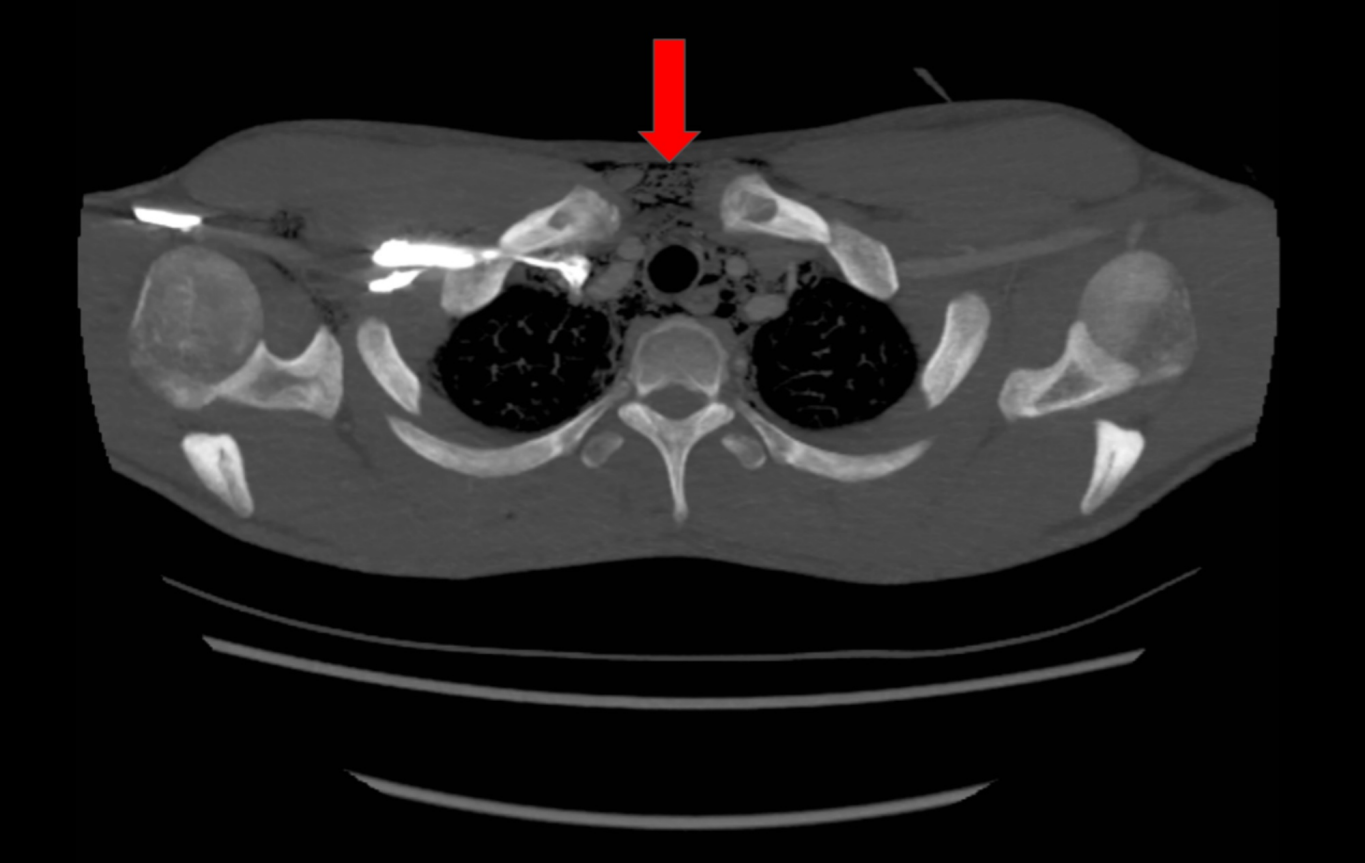
Transverse computed tomography (CT) showing air in the upper mediastinum and soft tissues of the thorax (red arrow)

**Figure 3:**
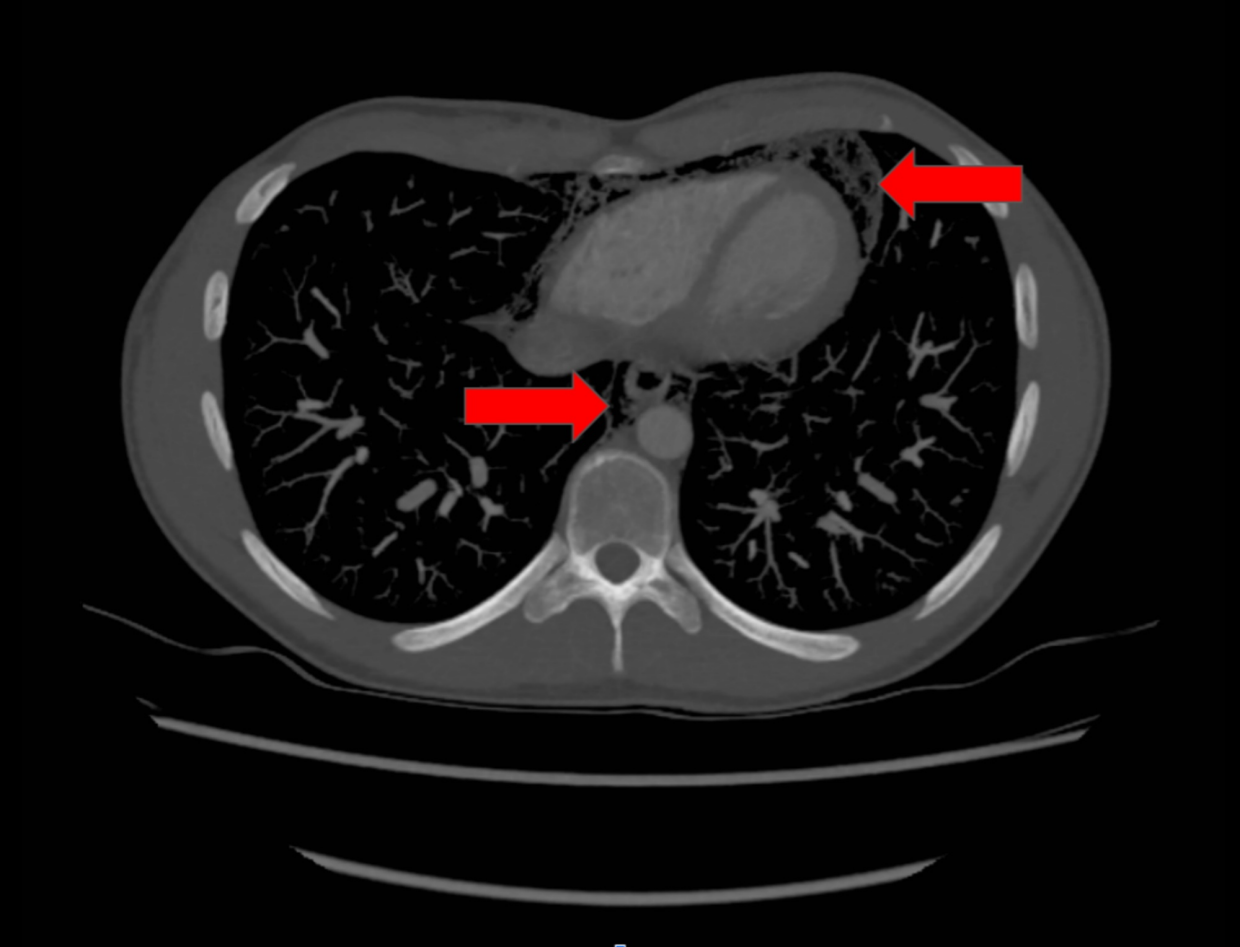
Transverse computed tomography (CT) showing lower air in the lower mediastinum and around the pericardium (red arrows)

**Figure 4:**
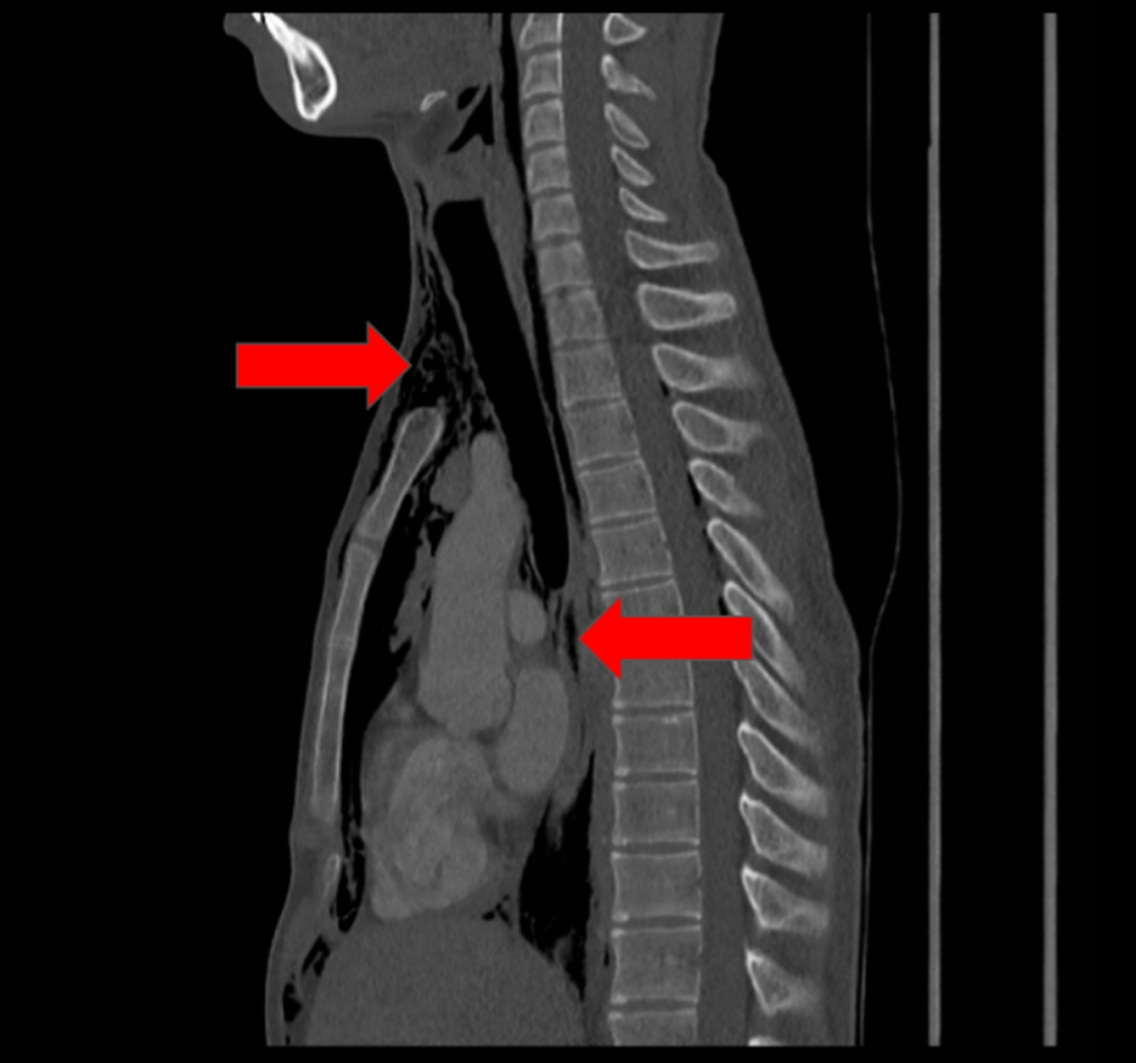
Sagittal computed tomography (CT) showing air in the mediastinum and soft tissues of the neck (red arrows)

The patient underwent a chest x-ray (CXR), which revealed widespread air in the soft tissues of the neck and thorax, including the mediastinum and chest wall (Figure 1). Contrast computed tomography (CT) (Figures 2-4) and contrast radiography were carried out to exclude oesophageal perforation or rupture of a laryngocele. This confirmed the CXR findings but did not reveal any further pathology.

The patient was admitted and observed as an inpatient for 48 hours. Respiratory rate, heart rate, blood pressure, and peripheral oxygen saturation were monitored and recorded every four hours. No intervention was required during his admission and the patient was discharged. Outpatient follow-up with CXR and clinical reassessment was arranged for one week later. Repeat CXR showed resolving changes. One year later, he remained well with no further medical problems or apparent long-term sequelae. 

## Discussion

The atypical presenting complaint and lack of a precipitating factor in this example make this an unusual presentation of this disease. A case series of 47 patients by Perna et al. found the acute onset of chest pain to be the most common presenting symptom and subcutaneous emphysema to be the most common sign on initial examination [8]. Other associated signs and symptoms include dyspnoea, odynophagia, and neck pain [9]. Hamman’s sign is a crunching sound heard synchronously with the heartbeat when auscultating the precordium and is usually considered pathognomonic [10].

Although a number of case reports and case series describing cases of spontaneous pneumomediastinum exist, this author is not aware of an instance of presentation to the emergency department with isolated neck swelling without any precipitating factors or any other associated symptoms. This is a rare but important differential of a patient presenting with neck swelling.

Recognition of pneumomediastinum is important due to the possible significant causes, as well as the potential for progression and deterioration requiring surgical intervention [11]. Patients presenting with signs and symptoms suggestive of pneumomediastinum often undergo imaging and investigation to confirm the diagnosis and to exclude secondary causes. Investigations mentioned in the literature include CXR, CT, bronchoscopy, and oesophagoscopy.

Management of these cases also continues to vary and the use of antibiotics, appropriate investigations, and surgical intervention have previously been discussed [12-14].

Systematic reviews of the literature and development of evidence-based guidelines may benefit the care of these patients by rationalising their care and limiting radiation exposure, thus preventing the overuse of antibiotics or surgical intervention and prevention of morbidity and mortality due to secondary complications.

## Conclusions

This is an unusual presentation of this rare disease. Following investigation to confirm the diagnosis and exclude sinister causes, the patient was managed conservatively with good effect. This corresponds with the usual course of this disease. Development of evidence and consensus-based guidelines may be helpful to standardise the management of patients presenting with suspected spontaneous pneumomediastinum.
